# Calcium Phosphate Growth at Electropolished Titanium Surfaces

**DOI:** 10.3390/jfb3020327

**Published:** 2012-04-25

**Authors:** Elnaz Ajami, Kondo-Francois Aguey-Zinsou

**Affiliations:** 1 School of Engineering and Materials Science, University of London, Queen Mary, London E1 4NS, UK; 2 School of Chemical Engineering, The University of New South Wales, Sydney NSW 2052, Australia; Email: f.aguey@unsw.edu.au

**Keywords:** surface treatment, electropolishing, titanium, hydroxyapatite, biomimetic, biomaterial

## Abstract

This work investigated the ability of electropolished Ti surface to induce Hydroxyapatite (HA) nucleation and growth *in vitro* via a biomimetic method in Simulated Body Fluid (SBF). The HA induction ability of Ti surface upon electropolishing was compared to that of Ti substrates modified with common chemical methods including alkali, acidic and hydrogen peroxide treatments. Our results revealed the excellent ability of electropolished Ti surfaces in inducing the formation of bone-like HA at the Ti/SBF interface. The chemical composition, crystallinity and thickness of the HA coating obtained on the electropolished Ti surface was found to be comparable to that achieved on the surface of alkali treated Ti substrate, one of the most effective and popular chemical treatments. The surface characteristics of electropolished Ti contributing to HA growth were discussed thoroughly.

## 1. Introduction

Titanium (Ti) and its alloys are popular biomaterials for load-bearing endosseous implants, because they exhibit a combination of favorable properties such as adequate mechanical strength, sufficient formability, low specific weight, excellent corrosion resistance and biocompatibility [1,2]. In terms of bone bonding, Ti can bond to bone [3], however the osteointegration does not occur well with unmodified Ti surfaces [2] and the bio-passive properties of Ti surfaces usually hinder the healing process [4]. To overcome such difficulties, there has been a growing interest in surface coating of Ti implants with Hydroxyapatite (HA), so that great osteoconductivity of HA combines with excellent mechanical properties of Ti. Popularity of HA among other calcium phosphate phases is because of its thermodynamic stability in biological conditions [5] and its existence in bone mineral phase. To date, various physical methods of HA coating on Ti surfaces have been investigated [1,6,7], however, the only commercially accepted method is plasma spraying. Major disadvantages associated with plasma spraying, *i.e*., high processing temperatures and poor integration of the coating with the metallic surface [8], have drawn attentions towards development of chemical coating methods based on mimicking biomineralization process [9]. In such biomimetic methods, the HA coating is produced by immersing the surface in a solution supersaturated with respect to HA (normally SBF) at a physiological temperature and pH, and a better HA integration onto metallic surface is achieved. However, kinetics of HA crystal nucleation and growth via biomimetic method might be very slow on the unmodified Ti surfaces. Therefore, many chemical surface treatment methods have been suggested to enhance the surface properties of Ti for accelerated HA nucleation, all of which aim to clarify the basic principles guiding the formation of HA from SBF on modified Ti surfaces. However, many contradictory observations are reported due to differences in the experimental conditions and thus it is difficult to draw a definitive conclusion on the mechanism of HA nucleation. Nonetheless, a general observation is that the surface charge plays a critical role in enhancing the mineralization of apatite. It has been found that at deprotonated Ti oxide layers (negatively charged), the adsorption of calcium ions and subsequent phosphate deposition would occur [10,11]. However, at positively charged Ti oxide surfaces, competitive adsorption between phosphate and chloride ions from SBF was found to hinder the growth of calcium phosphate phases [12]. For that reason, attempts have mainly focused on implanting negative ions at the surface of Ti by chemical or physical methods. Common chemical methods rely on the use of hydrogen peroxide [13], alkaline [7,14,15,16] or acidic solutions [17]. The titanate gel layer obtained after these treatments bears negatively charged surface groups under physiological conditions, which facilitate the precipitation of Ca^2+^ and further growth of apatite [15,18]. Functionalization of Ti surfaces with self-assembled monolayers (SAMs) also favors a mechanism whereby HA mineralization occurs at negatively charged surfaces only [19,20,21,22,23,24]. However, HA deposition has also been observed on positively charged SAMs, which indicates that, depending on experimental conditions (e.g., pH, temperature and ionic concentration of the SBF), various mineralization mechanisms with initial homogeneous and/or heterogeneous nucleation of apatite may occur [25].

Other methods to facilitate the deposition of HA are based on the direct implantation of ions such as Na^+^ [13,18], Ca^2+^ [26,27] and Mg^2+^ [28] onto Ti surfaces. For example, sodium titanate surfaces, implanted with Na^+^ ions, have been found to promote the nucleation and growth of HA from SBF [18,29]. Upon sodium titanate hydrolysis in SBF, the subsequent release of NaOH is believed to increase the pH locally, favor the deprotonation of surface hydroxyl groups and thus increase supersaturation level with respect to HA [18,29] at the solution/Ti interface [28,30,31]. Although these observations may contradict other findings with respect to negatively charged Ti surfaces, they also indicate that the nucleation of apatite may only proceed by the precipitation of Ca^2+^ ions first.

In this respect, the general trend observed with negatively charged Ti surfaces enhancing the mineralization of apatite has drawn our attention towards electropolished Ti surfaces. Upon electropolishing, Cl^−^ residues are usually left at the surface of Ti within a depth of 20 to 35 Å [32,33,34]. Such implanted ions should therefore enhance nucleation and growth of HA. To date, smooth and homogenous surfaces of electropolished Ti have only been used as well-defined starting points for subsequent surface treatments, such as anodic oxidation [33]. They have also been used as control surfaces to investigate the effect of surface roughness on cell behaviour or tissue reactions with Ti surfaces [35,36]. To the best of authors’ knowledge, not much attention has been paid to HA nucleating ability of electropolished Ti surface and therefore, this study aims to evaluate the ability of electropolished Ti surface to induce HA nucleation and growth via biomimetic method in SBF. In order to truthfully evaluate this ability, the behaviour of electropolished Ti substrate was also compared to that of Ti surfaces modified with conventional chemical methods including alkaline [14], hydrogen peroxide/hydrochloric acid mixtures [13], and *Piranha* [37] pre-treatments. To this aim, a range of experimental techniques including X-ray Photoelectron Spectroscopy (XPS), Energy-dispersive X-ray (EDX), X-ray diffraction (XRD) and Fourier Transform Infrared (FTIR) spectroscopy were used to fully characterize the physical and chemical properties of the modified Ti substrates. 

## 2. Results and Discussion

### 2.1. Surface Properties of the Pre-Treated Ti Substrates

**Figure 1:**
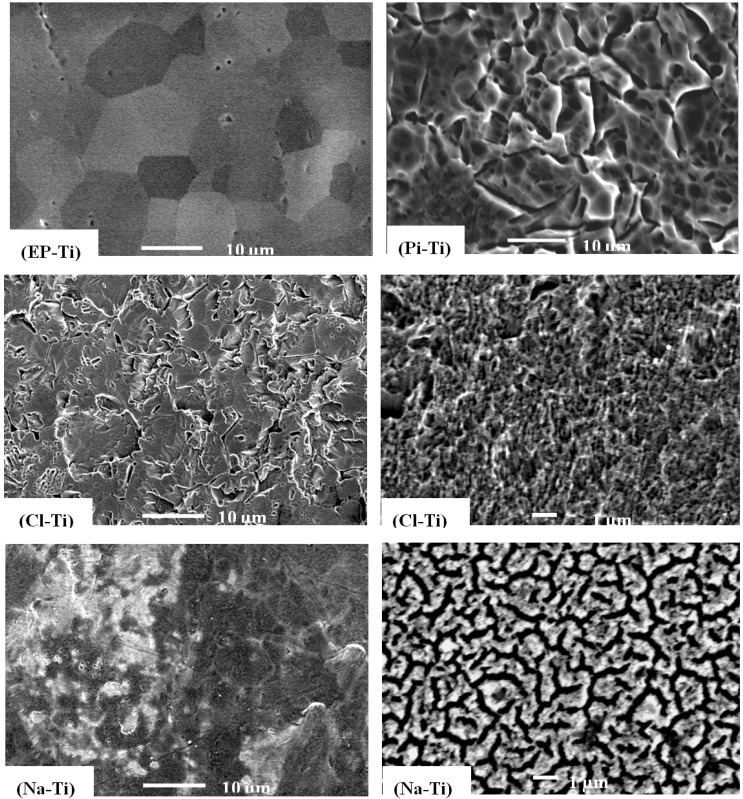
SEM images of EP-Ti, Pi-Ti, Cl-Ti (left) and Na-Ti (left) with scale bars of 10 µm. Magnified SEM images of Cl-Ti and Na-Ti on the right show the surface micro-porosity with scale bars of 1 μm.

The evolution of the morphology of the Ti substrates as function of pre-treatments was analyzed by SEM (Figure 1). Electropolishing resulted in a smooth surface with grain boundaries clearly observable (Figure 1, EP-Ti). However, considerable modifications of the surface morphology were observed for all the chemically treated Ti substrates. Hence, upon *Piranha* etching, large crevices (~2.5 µm wide) were formed at the surface of Ti (Figure 1, Pi-Ti). The use of HCl and H_2_O_2_ also resulted in the formation of a rough surface with a pore size of ~1 µm and crevices of ~2 µm long, but with additional micro-porosity (Figure 1, Cl-Ti) [13]. On the Ti surface treated with NaOH no crevices were observed, but a microstructure corresponding to a sodium titanate hydrogel was obtained (Figure 1, Na-Ti) [14,38,39]. 

**Figure 2:**
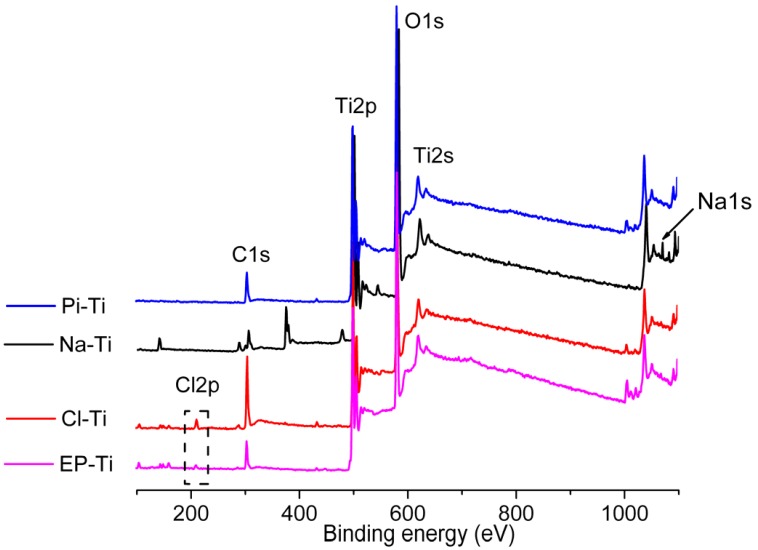
XPS wide-scan spectra of the electropolished and chemically treated Ti substrates before HA coating.

The surface chemistry of the substrates after pre-treatments was characterized by XPS. All the XPS survey spectra showed peaks related to Ti, O and C (Figure 2). The Auger peaks for Ti, O and C were also observed at higher binding energies. In addition, peaks related to Na1s at 1,072 eV on Na-Ti and Cl2p at 199.6 eV on both Cl-Ti and EP-Ti were detected. The Na1s peak was attributed to the formation of sodium titanate at the surface of Ti substrate pre-treated with NaOH [40]. Whereas, the Cl2p peak was assigned to the formation of TiOCl_x_ [41] at the surface of EP-Ti and Cl-Ti due to the adsorption of Cl^−^ or ClO_4_^−^ ions at the outermost surface as described previously [33,34,42]. It is noteworthy that no sulphur peaks were detected on substrates pre-treated with the *Piranha* solution (Pi-Ti). 

**Figure 3:**
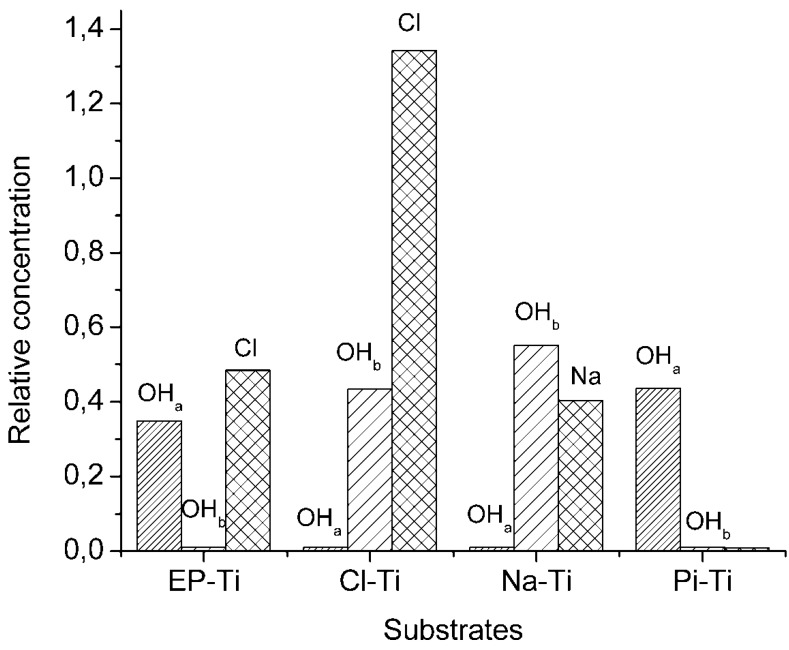
Relative concentration of hydroxyl groups (OH_a_ and OH_b_) and Na or Cl at the surface of the electropolished and chemically treated Ti substrates before HA coating. These concentrations were determined from the XPS results by taking the ratio of the peak area corresponding to a specific surface entity to that of the titanium peak.

XPS narrow-scan spectra of Ti2p (Supplementary Figure S1) for all of the substrates showed two main peaks at 458.8 and 464.4 eV, corresponding to TiO_2_ [43,44,45,46], and a weak peak at lower binding energies (~453.6 eV) only in the EP-Ti and Pi-Ti related to Ti^0^ [2]. The intensity of the latter was higher in EP-Ti compared to Pi-Ti, confirming a thinner oxide layer on EP-Ti, and the absence of such a peak in Na-Ti and Cl-Ti indicated that the thickness of the Ti oxide layer has increased beyond the penetration depth of the photoelectrons (>7 nm). Further characterization of the substrates in C1s region by XPS (Supplementary Figure S2) also revealed a significant level of carbon contamination due to CO_2_ and organic contaminants on all substrates, except on Pi-Ti. Furthermore, the XPS narrow-scan spectra in the O1s region (Supplementary Figure S3) revealed several peaks at 529, 530 and 531 eV, ascribed to the oxygen in bulk TiO_2_ (Ti-O) [31,47,48], surface acidic (OH_a_) and basic (OH_b_) hydroxyl groups, respectively [31,43,49]. The OH_a_ and OH_b_ correspond to doubly and singly coordinated hydroxyls, respectively [50], and the density of these hydroxyl groups was found to decrease in the following order: Na-Ti > Pi-Ti~Cl-Ti > EP-Ti (Figure 3). This is in agreement with previously reported data showing the highest density of hydroxyl groups on Ti substrates pre-treated with NaOH [51]. It should also be highlighted that EP-Ti and Pi-Ti surfaces showed OH_a_ only, while on Cl-Ti and Na-Ti, only OH_b_ were found (Figure 3). 

### 2.2. Ti Substrates upon CaP Coating

#### 2.2.1. Physical Properties of the CaP Coating

**Figure 4:**
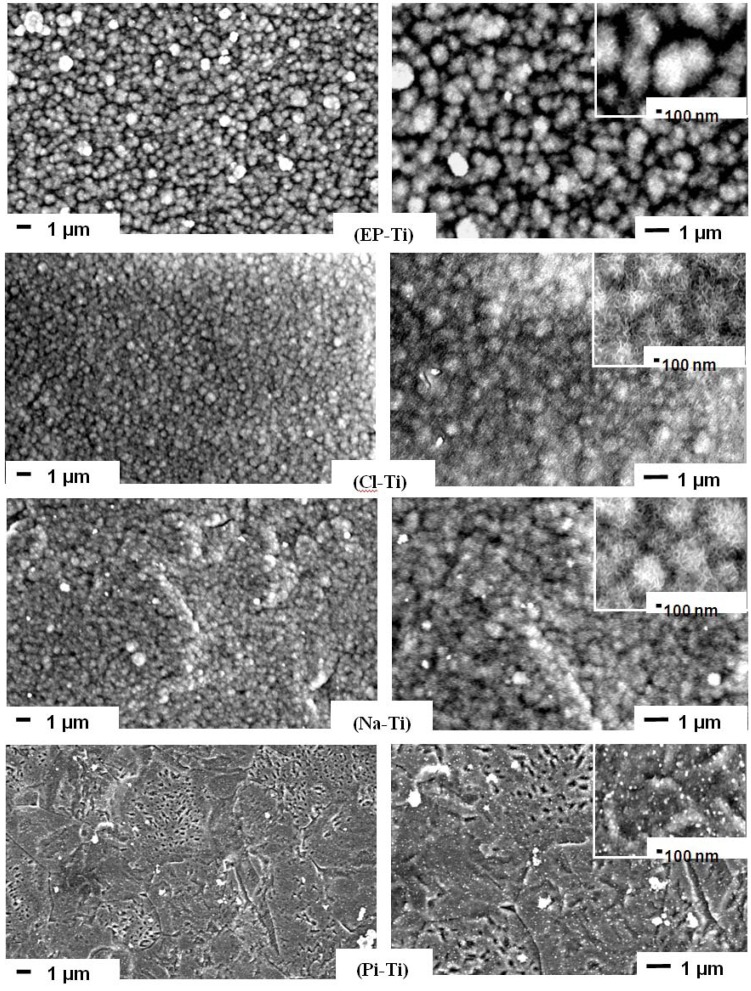
SEM images of EP-Ti, Cl-Ti, Na-Ti and Pi-Ti after 14 days immersion in 1.5 SBF. Images on the right are magnified.

The pre-treated substrates were immersed in 1.5 SBF and the formation of a CaP coating was characterized after 14 days of immersion. Except for Pi-Ti, relatively dense CaP coatings were found by SEM at the surface of all pre-treated substrates (Figure 4). The CaP films comprised globular structures (1 to 0.5 µm) consisting of dense plate-like crystals (Supplementary Figure S4) as a result of a three-dimensional growth process [13,52,53]. These globular structures are typical to biomimetic methods [38,54,55,56,57,58,59,60,61,62] and are observed in the bone cement line matrix interlocking with the surface of the modified titanium oxide at the bone-implant interface [63]. On the surface of Pi-Ti, only small particles of CaP (~50 nm in diameter) were found and large globular structures of CaP could only be observed after 1 month of immersion (Supplementary Figure S5). Accordingly, it can be speculated that the surface of Pi-Ti induces CaP with slower growth kinetics. Such a slow kinetics on Pi-Ti is neither attributed to the reduced thickness of oxide layer on this surface nor to the nature of surface hydroxyl groups. The reason is that when Pi-Ti was compared to EP-Ti, the XPS Ti2p spectra (Supplementary Figure S1) proved a thicker oxide layer on Pi-Ti compared to EP-Ti, and as shown in Figure 3, both had acidic hydroxyl groups. Therefore, it is believed that lack of charged groups on the surface of Pi-Ti is the reason for its slower kinetics in inducing CaP growth as compared to other surfaces. 

Comparing the coating on Cl-Ti and EP-Ti, it is observed that the coating on EP-Ti is less dense than that on Cl-Ti and the underlying Ti substrate is exposed in between globular structures on EP-Ti (as indicated by arrows on the magnified inset image of EP-Ti in Figure 4). This could be explained by lower density of surface OH on EP-Ti compared to Cl-Ti as confirmed by XPS spectra in O1s region. Moreover, a sharper Cl2p that is observed on XPS survey spectrum of Cl-Ti compared to EP-Ti shows that there are more Cl^−^ ions contributing to the mineralization process, thus resulting in a denser coating on Cl-Ti compared to EP-Ti.

**Figure 5:**
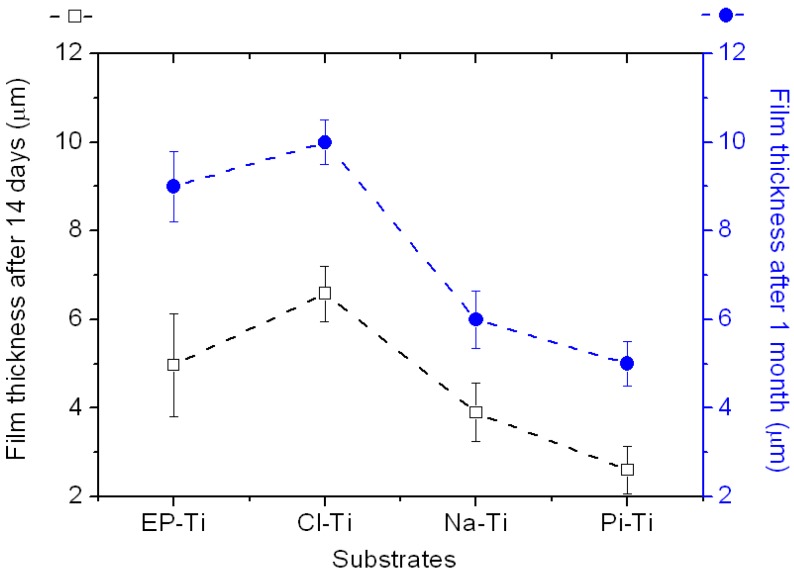
Evolution of the CaP film thickness on all substrates after immersion in 1.5 SBF for 14 days and 1 month. Film thickness was measured on three different locations with cross-sectional SEM imaging. The dashed lines are guides to the eye.

Further characterization of the film thickness revealed that different growth regimes occur at various Ti substrates, with Cl-Ti and EP-Ti showing the thickest film (~9.5 µm) after 1 month of immersion in 1.5 SBF (Figure 5). It is noteworthy that globular films have formed on all surfaces regardless of the initial surface roughness. This suggests that heterogeneous nucleation of CaP on Ti had happened immediately after immersion in 1.5 SBF and did not depend on the surface topography in agreement with previous reports [57]. However, this statement is only valid for *in vitro* surface-induced mineralization of CaP on Ti surfaces and it is not applicable to bone bonding onto Ti implant surfaces. It is very well understood that implant surfaces with complex microtopography, and not the smooth surfaces, render surfaces bone bonding [64]. In terms of *in vitro* surface-induced mineralization, it can be speculated that the surface topography would only influence the mechanical stability of final CaP film and not the initial surface-induced formation of CaP nuclei. This is because a more adherent CaP coating will be formed on rough surfaces due to increased interfacial adhesion strength between the coating and Ti surface [57,65,66]. 

**Figure 6:**
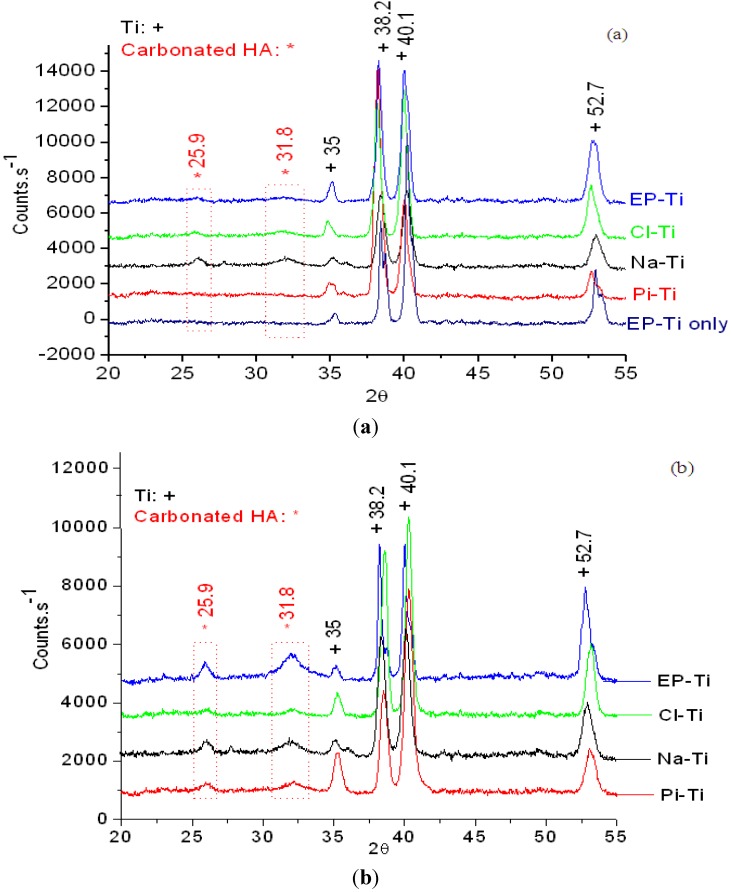
XRD patterns of the CaP film formed on the different substrates after (**a**) 14 days and (**b**) 1 month immersion in 1.5 SBF.

The crystallinity of the films was investigated by XRD after immersion in 1.5 SBF for 14 days (Figure 6a). All substrates showed peaks at 2θ = 35, 38.2, 40.1 and 52.7° related to the (100), (002), (101) and (102) planes of Ti, respectively [13,18,28,55,67,68]. Once more, except for Pi-Ti, all substrates showed peaks characteristic of a carbonated HA, *i.e*., at 2θ = 25.9 and 31.8°. The former peak corresponds to the overlapping of the (201) and (002) planes of carbonated HA, while the latter is related to the overlapping of the (211), (112) and (300) reflections [28,55,67,69,70,71,72]. The additional peak at 2θ = 35.5–36° only appearing on the XRD patterns of the Na-Ti and Pi-Ti was attributed to the superposition of the carbonated HA or tricalcium phosphate peak. The broadness of the HA peaks also indicates very small crystal sizes and/or the orientation of crystals at different directions. The absence of these reflexes (and the low intensity of the peaks) is probably due to the thinness of the CaP film [19]. Moreover, the intensity of the peak at 2θ = 25.9° is equal to that of 2θ = 31.8° in the patterns, while it should normally be less than a quarter for the carbonated HA. This could be due to the reflection of other crystalline phases, such as crystalline dicalcium phosphate with an XRD peak at 2θ = 23.2° corresponding to (040) plane [73] and tricalcium phosphate with an XRD peak at 2θ = 25.7° corresponding to the (10 10) plane [74,75]. Both of these peaks may superimpose on the carbonated HA peak at 2θ = 25.9° resulting in a higher intensity peak.

After 1 month of immersion in 1.5 SBF, the intensity of both HA peaks at 25.9 and 31.8° was found to increase considerably on the EP-Ti and Pi-Ti, which confirms the formation of a thicker HA film and/or better crystallinity of the HA phase (Figure 6b). Intensity of the peaks related to HA on other substrates did not show much change. Further determination of the HA crystal size using the Scherrer formula [76] revealed similar crystallite sizes on all substrates expect for EP-Ti (Table 1), where HA crystallite size was found to double from 3.5 after 14 days to 6.4 nm after 30 days of immersion in 1.5 SBF. While the formation of a very thin film on the Pi-Ti compared to other substrates confirms fast kinetics of CaP film growth on the charged Ti surfaces, the formation of thickest film on the Cl-Ti proves enhanced CaP film growth on negatively charged surfaces in agreement with previous observations [23]. 

**Table 1:** Crystal size of HA formed at the surface of the substrates after 14 days and 1 month in 1.5 SBF.

HA crystallite size (±1.0 nm)	Na-Ti	EP-Ti	Pi-Ti	Cl-Ti
after 14 days	5.5	3.5	-	4.4
after 30 days	6.0	6.4	5.2	5.2

#### 2.2.2. Chemical Composition of the Coating

**Figure 7:**
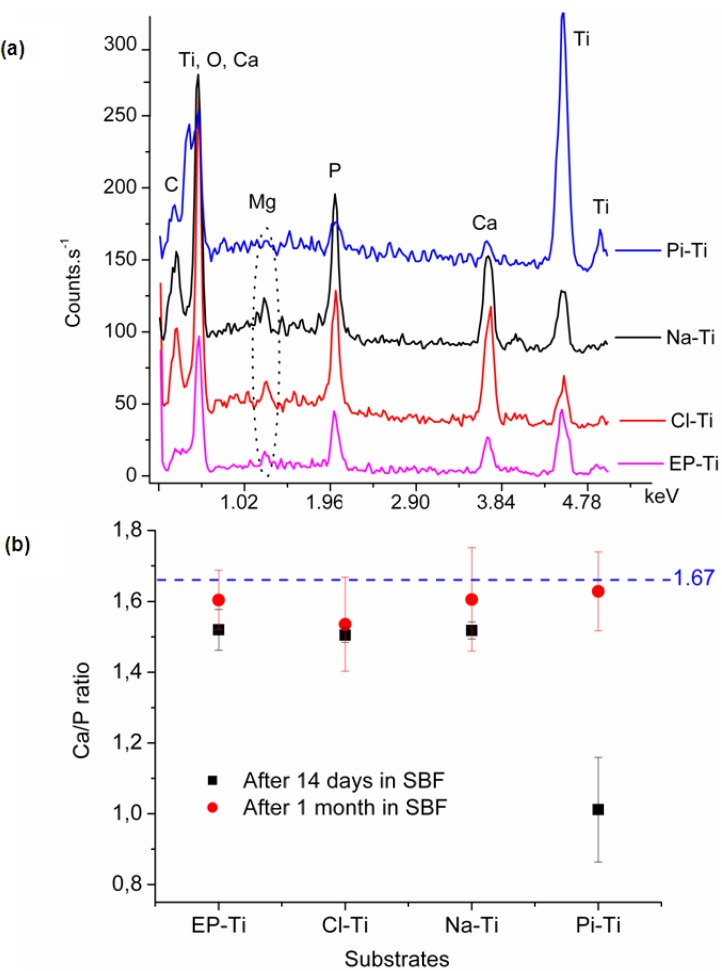
(**a**) Typical EDS spectra obtained on all substrates after 14 days immersion in 1.5 SBF; (**b**) Ca/P ratio of the CaP film as determined by EDS analysis on the substrates after 14 days and 1 month immersion in 1.5 SBF.

Upon EDX analysis, sharp peaks related to Ca and P were observed confirming the formation of CaP phases on all surfaces. Depending on the thickness of the film, peaks of different intensities related to Ti were also observed (Figure 7a). Furthermore, additional peaks corresponding to C and Mg were detected. The C peak was either due to the surface carbon contamination or carbonate ions in the lattice structure of CaP crystals. Similarly, it is believed that Mg^2+^ ions had partially substituted Ca^2+^ ions in the CaP lattice structure. Presence of similar traces of Mg^2+^ in the EDX and XPS spectra of the CaP film formed at alkali treated Ti surfaces also supports such a hypothesis[E1] [40,77].

Further analysis of the EDX results after 14 days of immersion in 1.5 SBF showed that the Ca/P ratio on Pi-Ti is much lower than that of synthetic HA (*i.e*., ~1.67) and is related to Ca/P of dicalcium phosphate (Figure 7b). This small Ca/P ratio could be either due to the low film thickness along with the low detection limit of the instrument, or an inadequate surface chemistry of Pi-Ti in enabling Ca^2+^ adsorption. However, on Na-Ti, EP-Ti and Cl-Ti, a Ca/P ratio of 1.50 was found and could be related to a Ca-deficient HA phase.

After 30 days of immersion in 1.5 SBF, the Ca/P ratio was found to be close to that of synthetic HA, *i.e*., 1.67 (Figure 7b). The Ca/P ratio on all substrates was mainly around 1.55, which corresponds to a Ca-deficient HA. This could be due to a partial substitution of Ca^2+^ by Mg^2+^ ions or substitution of PO_4_^3−^ by CO_3_^2−^ in the crystal lattice structure, as previously discussed. Furthermore, it can be concluded that once the initial growth regime has been completed and an equilibrium state has been achieved, the Ca/P ratio is relatively the same on all substrates regardless of the early stages of the nucleation process. This would indicate similar growth mechanisms on all substrates after the initial nucleation and growth of the apatite. 

With respect to the deposition mechanism of CaP, the behaviour of the Na-Ti substrate in SBF has been explained by Kim *et al*. [15,51,78]. According to their results, the release of Na^+^ ions from the substrate induces a pH increase at the substrate/SBF interface, which leads to the deprotonation of OH_b_ groups at the Ti surface. Therefore, the adsorption of Ca^2+^ ions, which are initiators of the nucleation and growth of apatite, is enhanced. However, in the case of EP-Ti and Cl-Ti, the nucleation and growth mechanism of apatite may significantly differ, as the additional Cl^−^ at EP-Ti and Cl-Ti may tend to stay on the surface due to the high concentration of Cl^−^ ions in 1.5 SBF. These adsorbed Cl^−^ may facilitate the electrostatic adsorption of Ca^2+^ and thus trigger the heterogeneous precipitation of a CaP phase. This phenomenon could also be enhanced by the deprotonation of OH_a_ at the surface of EP-Ti, since a pKa ~2.9 would be expected for the OH_a_ populating the surface of EP-Ti [79]. However, the role of OH_a_ in comparison to that of Cl^−^ should be minimized, since the initial deposition of CaP phases at the Pi-Ti surface comprising only OH_a_ surface groups is slow (Figure 3 and Figure 5). The implantation of Cl^−^ ions at the surface of EP-Ti would therefore explain the good CaP induction ability observed after electropolishing in comparison to conventional chemical treatments. 

**Figure 8:**
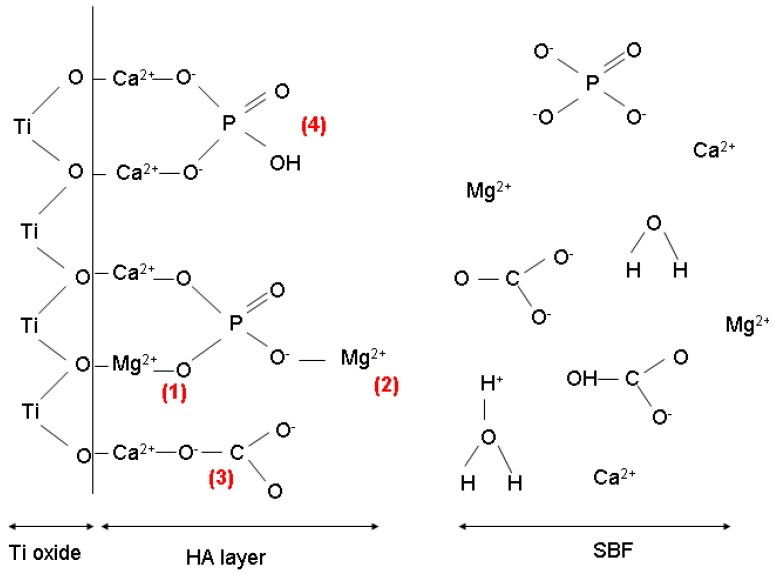
Proposed mechanism of HA nucleation in the presence of Mg^2+^ and CO_3_^2−^ ions at the Ti/SBF interface. (**1**) Mg^2+^ substitutes Ca^2+^ in the lattice structure or adsorbs directly on the surface; (**2**) Mg^2+^ forms magnesium phosphate and inhibits further adsorption of Ca^2+^; (**3**) CO_3_^2−^ substitutes PO_4_^3−^; (**4**) Protonated PO_4_^3−^ ions after adsorption at calcium sites.

Other ions in SBF, in particular Mg^2+^ and CO_3_^2−^, have also influenced the nucleation and growth of CaP, its composition and possibly its kinetics of growth, as evidenced by EDX and XRD analysis. In fact, Mg^2+^ and CO_3_^2−^ ions can compete for adsorption at the surface of Ti and form complexes preventing further CaP precipitation, as highlighted in Figure 8 (mechanisms 1, 2 and 3) [80,81]. For example, traces of Mg^2+^ ions have been shown to reduce the overall rate of CaP crystallization and delay the transformation of amorphous CaP to apatite phases [82]. Furthermore, the formation of magnesium phosphates in solution would reduce the amount of phosphate ions available to form CaP phases [80]. As a consequence, high concentrations of Mg^2+^ ions at the substrate/solution interface would favor the heterogeneous nucleation of poorly crystallized CaP phases and hinder the growth of HA crystals. This inhibitory effect of Mg^2+^ and CO_3_^2−^ ions on the growth of HA could explain the small crystal size of HA (~4–6 nm) obtained from the XRD patterns in this work. It could also lead to strong binding of HA to the substrate [81]. 

**Figure 9:**
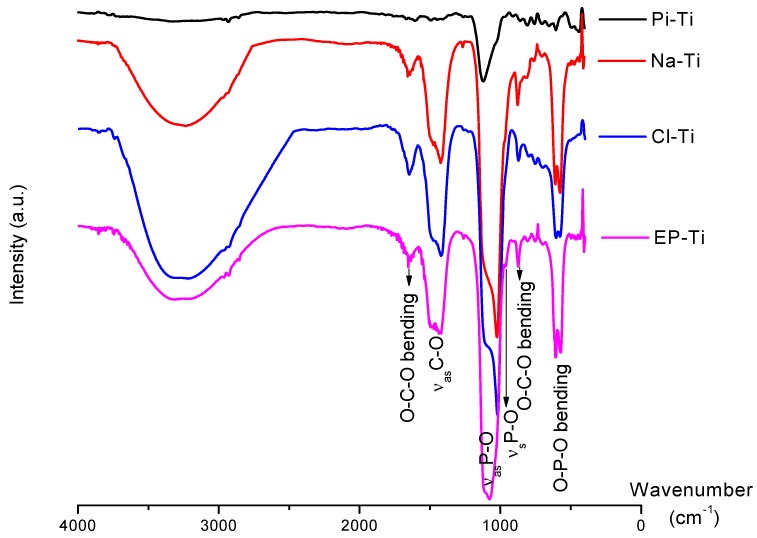
FTIR spectra of the substrates after 14 days immersion in 1.5 SBF. The possible shifts in the peaks between 1,015–1,120 cm^−1^ compared to those of stoichiometric HA are due to the presence of CO_3_^2−^, HPO_4_^2−^ and crystal imperfections.

In order to determine the chemical formula of the carbonated HA formed on the substrates, further characterizations were carried out by FTIR (Figure 9 and Table 2). The obtained spectra, and the symmetric and asymmetric vibrations at 1,015–1,120 and 920 cm^−1^ confirmed the apatitic nature of the CaP films formed on all substrates. Furthermore, the vibration observed at 872 cm^−1^, characteristic of a B-type apatite (*i.e*., substitution of PO_4_^3−^ by CO_3_^2−^ ions) [83,84], revealed that the apatite obtained on all substrates was partially of type-B. In fact, the frequency observed for C-O asymmetric stretching at 1,415–1,490 cm^−1^ is too high to correspond to a B-type apatite and is more similar to those usually observed for A-type apatite (*i.e*., substitution of OH^−^ by CO_3_^2−^ ions) [85]. The absence of the apatite OH stretching peaks at 630 cm^−1^ also partly confirmed the substitution of A-sites by the carbonate ions [70,86,87,88]. However, the characteristic IR peak for the A-type apatite, *i.e*., at 1,545 cm^−1^ [89], was not observed. Therefore, an AB-type apatite had most likely formed on all the Ti substrates in agreement with previous investigations showing the formation of an AB-type apatite at neutral pH (7.4 for the 1.5 SBF used in the present study) [83,85].

**Table 2:** Assignments of FTIR peaks for PO_4_^3−^ and CO_3_^2−^ vibrations on the spectra obtained and display on Figure 10.

FTIR peaks related to PO_4_^3−^	Wavenumber (cm^−1^)
*ν*_4_: O-P-O bending	Doublet peaks at 571 and 604
*ν*_1_: P-O symmetric stretching	960
*ν*_3_: P-O asymmetric stretching	doublet peaks between 1,015–1,120
**FTIR peaks related to CO_3_^2−^**	**Wavenumber (cm^−1^)**
*ν*_2_: O-C-O out-of-plane bending	872
*ν*_3_: C-O asymmetric stretching	doublet peaks between 1,415–1,490
*ν*_3_: O-C-O bending	Between 1,640–1,650

Based on these results, a chemical formula for the obtained HA film is suggested considering the fact that trivalent anionic phosphate sites can only be occupied by bivalent hydrogen phosphates or carbonate anions, and monovalent hydroxyl sites can only be substituted by carbonate ions. Furthermore, taking into account that calcium sites can be occupied by other cations and can accept a maximum of two vacant sites [90], the AB-type apatite formed may have the following formula: Ca_10−x−y_Mg_y_(HPO_4_)_x−z_(CO_3_)_z_(PO_4_)_6−x_(OH)_2−x−w_(CO_3_)_w/2_, 0 ≤ x,y,z,w ≤ 1.

## 3. Experimental Section

All chemicals including methanol (AR grade), perchloric acid (ACS grade, ≥69%), 1-butanol (ACS grade, ≥99.5%), hydrogen peroxide 30 wt.% in water (ACS reagent), hydrochloric acid (GPR grade, 38%), sulphuric acid (AR grade, >97.5%), ethanol (GPR grade), acetone (GPR grade) were purchased from Sigma and used as received. All chemicals for SBF preparation (listed in Table 3) were also purchased from Sigma (ACS reagent grade).

**Table 3:** Composition of 1.5 SBF listed in sequence of dissolution.

#	Chemicals	Amount (g) for 1.5 SBF in 1L of water
1	NaCl	10.806
2	NaHCO_3_	1.472
3	Na_2_CO_3_	4.072
4	KCl	0.45
5	K_2_HPO_4_.3H_2_O	0.476
6	MgCl_2_.H_2_O	0.622
7	HEPES	23.856
8	CaCl_2_	0.586
9	Na_2_SO_4_	0.144
10	1M-NaOH	3 mL

Commercially pure Ti plates were purchased from Goodfellow and cut to 1 × 2.5 cm^2^. Platinum/Rhodium foil (Pt90/Rh10) of 25 × 25 × 0.125 mm^3^ size was purchased from Goodfellow and used as the cathode in the electropolishing unit. 

### 3.1. Electropolishing

Before electropolishing, the as-received Ti substrates were first polished with a #1000 and then a #2400 silicon carbide grinding paper to remove the native oxide layer. The polished substrates were cleaned by sonication in distilled water, ethanol and then acetone, and finally dried in air. 

**Figure 10:**
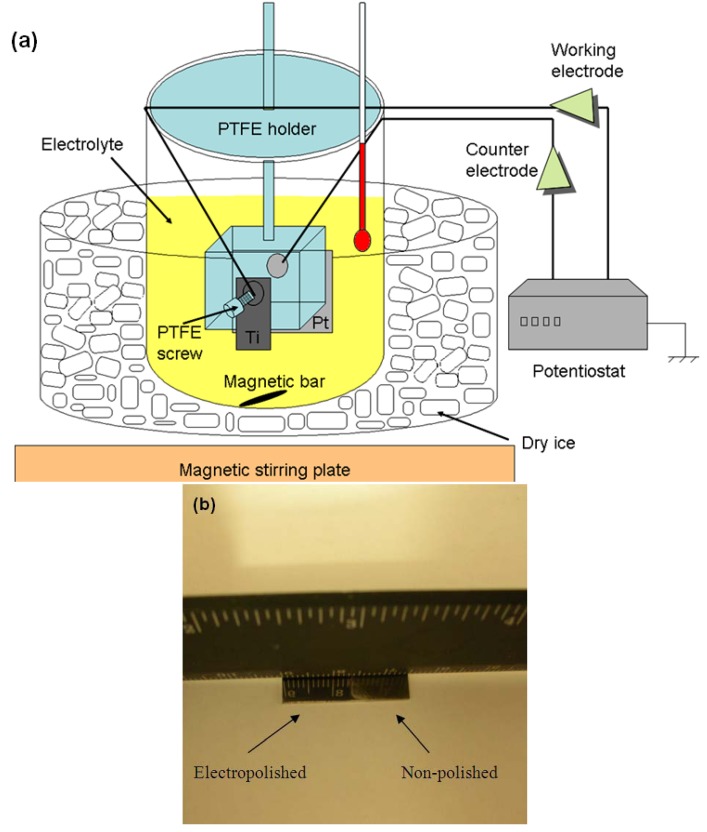
(**a**) Schematic of the home made electropolishing unit; (**b**) Photo of a substrate with the electropolished surface at half length. The reflection of the ruler on the electropolished part is clearly seen.

The electrochemical cell was home-made (Figure 10a) and a mixture of 54 mL methanol, 25 mL 1-butanol and 6 ml perchloric acid was used as the electrolyte. Electropolishing was conducted at −30 °C, under an anodic potential of 9.5 V for 5 min. Vigorous agitation was maintained throughout the process in order to obtain a homogenously electropolished surface [91]. Once electropolished, the Ti substrate was sonicated in methanol, ethanol and water, and finally dried under blowing Ar before storage under Ar (substrate denoted EP-Ti). The electropolished surface had a mirror finish as shown in Figure 10b.

### 3.2. Conventional Surface Treatments

The as-received Ti substrates were cleaned by sonication in water, ethanol and acetone. Three conventional chemical treatments were used to modify the surface chemistry of the Ti substrates: (a) immersion in a 2:1 *Piranha* solution (*v*:*v*, H_2_SO_4_: H_2_O_2_) at 100 °C for 10 min (substrate denoted Pi-Ti); (b) soaking in a 5 M sodium hydroxide (NaOH) solution at room temperature for 24 h (substrate denoted Na-Ti); (c) dipping in a mixture of 8.8 M H_2_O_2_ + 0.1 M HCl at 80 °C for 1 h (substrate denoted Cl-Ti). All substrates were rinsed with a copious amount of distilled water after chemical treatments and then blow dried with Ar.

### 3.3. CaP Coating

For the nucleation and growth of HA, a “revised” SBF solution was used (see Table 3 for composition) [92]. In order to decrease the immersion time required to start the process of mineralization in SBF, a 1.5 SBF was used. Using 1.5 SBF, and not 1 SBF, is not conflicting with the report of Kokubo *et al*. [93], as the scope of the current study is not to evaluate the *in vivo* bone bioactivity of a material, and the SBF is used as a supersaturated solution with calcium and phosphate to study the effect of surface charge in the nucleation and growth of calcium phosphate phases *in vitro*. 1.5 SBF was prepared by dissolving the chemicals listed in Table 3 in sequence in 1,000 mL of high purity water in a polyethylene beaker at 36.5 °C under Ar. The solution was buffered at pH = 7.4 at 36.5 °C and the stock solution showed no precipitations after 1 month at 4 °C. Three substrates per treatment group were prepared and suspended with a cotton thread individually in 20 mL of 1.5 SBF at 36.5 °C in a polyethylene bottle under Ar to limit CO_2_ dissolution. The sealed bottles were stirred throughout the experiments and the solution was refreshed every 3 days. Once the substrates were removed from the solution, they were washed gently with high purity water and dried under flowing Ar. The substrates were stored under Ar until being characterized. 

### 3.4. Characterization Methods

The chemical composition of Ti surfaces before CaP coating was determined by X-ray Photoelectron Spectroscopy (XPS) using a VG-Microtech Mutilab 3000 instrument. The XPS measurements were performed at 10^−7^ Pa with an Al Kα source at 150 W.

The surface morphology of Ti substrates before and after CaP coating was further characterized using a Field Emission Scanning Electron Microscope (FE-SEM) JEOL 6301 operated at 5 kV. Chemical composition of the surface with CaP coating was gained by Energy-dispersive X-ray (EDX) analysis. Further characterization of the chemical composition of the HA coating was done by Infrared spectroscopy. A Digilab Fourier Transform Infrared (FTIR) spectrophotometer equipped with a diffuse reflectance accessory was used. The spectra were collected with 100 scans at a resolution of 4 cm^−1^. The crystallographic structure of the CaP film was analyzed using a Bruker thin film X-ray Diffractometer (XRD) equipped with a Cu Kα radiation source. The starting incident angle used was 5°.

## 4. Conclusions

The formation of bone-like apatite film was achieved on the surface of all chemically treated substrates despite differences in mechanism of HA growth and kinetics, with an enhancement of HA mineralization at negatively charged surfaces. Hence, the electropolished Ti surface was found to be capable of inducing bone-like apatite and the formation of an AB-type carbonated HA layer was confirmed after only a few days of immersion of the substrate in SBF. The enhanced HA induction ability of electropolished Ti is believed to result from residual surface Cl^−^ formed during electrochemical process. By providing sites to the adsorption of Ca^2+^, such surface Cl^−^ would facilitate the nucleation and growth of HA. Therefore, electropolishing of Ti surfaces could be an alternative route to chemical modification techniques for facilitated growth of apatite on Ti surfaces via biomimetic methods. 

## Supplementary Files

Supplementary File 1 PDF-Document (PDF, 621 KB)

## References

[1] Liu X., Chu P.K., Ding C. (2004). Surface modification of titanium, titanium alloys, and related materials for biomedical applications. Mater. Sci. Eng. A.

[2] Brunnette D.M., Tengvall P., Textor M., Thomsen P. (2001). Titanium in Medicine. Materials Science, Surface Science, Engineering, Biological Responses and Medical Applications.

[3] Gross K.A., Berndt C.C. (1998). Thermal processing of hydroxyapatite for coating production. J. Biomed. Mater. Res..

[4] Steinemann S.G. (1998). Titanium—The materials of choice?. Periodontology 2000.

[5] Koutsopoulos S. (2002). Kinetic study on the crystal growth of hydroxyapatite. Langmuir.

[6] Blackwood D.J., Seah K.H.W. (2010). Galvanostatic pulse deposition of hydroxyapatite for adhesion to titanium for biomedical purposes. Mater. Sci. Eng. C.

[7] Ueda M., Ikeda M., Ogawa M. (2009). Chemical-hydrothermal combined surface modification of titanium for improvement of osteointegration. Mater. Sci. Eng. C.

[8] Sun L., Berndt C.C., Gross K.A., Kucuk A. (2001). Material fundamentals and clinical performance of plasma-sprayed hydroxyapatite coatings: A review. J. Biomed. Mater. Res..

[9] Habibovic P., Barrere F., van Blitterswijk C.A., de Groot K., Layrolle P. (2002). Biomimetic hydroxyapatite coating on metal implants. J. Am. Ceram. Soc..

[10] Stumm W. (1992). Chemistry of the Solid-Water Interface: Processes at the Mineral-Water and Particle-Water Interface in Natural Systems.

[11] Li P., Zhang F. (1990). The electrochemistry of a glass surface and its application to bioactive glass in solution. J. Non Cryst. Solids..

[12] Calvert P., Mann S. (1997). The nagtive side of crystal growth. Nature.

[13] Wang X., Hayakawa S., Tsurub K., Osaka A. (2002). Bioactive titania gel layers formed by chemical treatment of Ti substrate with a H_2_O_2_/HCl solution. Biomaterials.

[14] Lee B.H., Kim Y.D., Shin J.H., Lee K.H. (2002). Surface modification by alkali and heat treatments in titanium alloys. J. Biomed. Mater. Res..

[15] Kim H.M., Miyaji F., Kokubo T., Nakamura T. (1996). Preparation of bioactive Ti and its alloys via simple chemical surface treatment. J. Biomed. Mater. Res..

[16] Wei M., Kim H.M., Kokubo T., Evans J.H. (2002). Optimising the bioactivity of alkaline-treated titanium alloy. Mater. Sci. Eng. C.

[17] Wen H.B., de Wijn J.R., Liu Q., de Groot K. (1997). A simple method to prepare calcium phosphate coatings on Ti6Al4V. J. Mater. Sci. Mater. Med..

[18] Pham M.T., Maitz M.F., Matz W., Reuther H., Richter E., Steiner G. (2000). Promoted hydroxyapatite nucleation on titanium ion-implanted with sodium. Thin Solid Films.

[19] Majewski P.J., Allidi G. (2006). Synthesis of hydroxyapatite on titanium coated with organic self-assembled monolayers. Mater. Sci. Eng. A.

[20] Huang S., Zhou K., Liu Y., Huang B. (2003). Controlled crystallization of hydroxyapatite under hexadecylamine self-assembled monolayer. Trans. Nonferrous Met. Soc. China.

[21] Zhu P., Masuda Y., Koumoto K. (2001). A novel approach to fabricate Hydroxyapatite coating on titanium substrate in an aqueous solution. J. Ceram. Soc. Jpn..

[22] Tanahashi M., Matsuda T. (1997). Surface functional group dependence on apatite formation on self-assembled monolayers in a simulated body fluid. J. Biomed. Mater. Res..

[23] Li P., Ohtsuki C., Kokubo T., Nakanishi K., Soga N., de Groot K. (1994). The role of hydrated silica, titania, and alumina in inducing apatite on implants. J. Biomed. Mater. Res..

[24] Ajami E., Aquey-Zinsou K.F. (2011). Formation of OTS self-assembled monolayers at chemically treated titanium surfaces. J. Mater. Sci. Mater. Med..

[25] Zhu P., Masuda Y., Koumoto K. (2004). The effect of surface charge on hydroxyapatite nucleation. Biomaterials.

[26] Hanawa T., Kamiura Y., Yamamoto S., Kohgo T., Amemiya A., Ukai H., Murakami K., Asaoka K. (1997). Early bone formation around calcium-ion-implanted titanium inserted into rat tibia. J. Biomed. Mater. Res..

[27] Xie Y., Liu X., Chu P.K., Ding C. (2006). Nucleation and growth of calcium-phosphate on Ca-implanted titanium surface. Surf. Sci..

[28] Wan Y.Z., Huang Y., He F., Wang Y.L., Zhao Z.G., Ding H.F. (2006). Effect of Mg ion implantation on calcium phosphate formation on titanium. Surf. Coating Tech..

[29] Maitz M.F., Pham M.T., Matz W., Reuther H., Steiner G., Richter E. (2002). Ion beam treatment of titanium surfaces for enhancing deposition of hydroxyapatite from solution. Biomol. Eng..

[30] Hanawa T. (1999). *In vivo* metallic biomaterials and surface modification. Mater. Sci. Eng. A.

[31] Feng B., Chen J.Y., Qi S.K., He L., Zhao J.Z., Zhang X.D. (2002). Carbonate apatite coating on titanium induced rapidly by precalcification. Biomaterials.

[32] Jobin M., Taborelli M., Descouts P. (1993). Surface properties of electropolished titanium and vanadium. Appl. Surf. Sci..

[33] Lausmaa J., Kasemo B., Mattsson H., Odelius H. (1990). Multi-technique surface characterization of oxide films on electropolished and anodically oxidized titanium. Appl. Surf. Sci..

[34] Mathieu J.B., Mathieu H.J., Landolt D. (1978). Electropolishing of titanium in perchloric acid—Acetic acid solution. J. Electrochem. Soc..

[35] Larsson C., Thomsen P., Lausmaa J., Rodahl M., Kasemo B., Ericson L.E. (1994). Bone response to surface modified titanium implants: Studies on electropolished implants with different oxide thicknesses and morphology. Biomaterials.

[36] Meredith D.O., Eschbach L., Wood M.A., Riehle M.O., Curtis A.S.G., Richards R.G. (2005). Human fibroblast reactions to standard and electropolished titanium and Ti-6Al-7Nb, and electropolished stainless steel. J. Biomed. Mater. Res. A.

[37] Lewandowska M., Wlodkowska M., Olkowski R., Roguska A., Polak B., Pisarek M., Lewandowska-Szumiel M., Kurzydłowski K.J. (2007). Chemical surface modifications of titanium implants. Macromol. Symp..

[38] Fatehi k., Moztarzadeh F., Solati-Hashjin M., Tahriri M. (2008). *In vitro* biomimetic deposition of apatite on alkaline and heat treated Ti6Al4V alloy surface. Bull. Mater. Sci..

[39] Chen Y., Zheng X., Ji H., Ding C. (2007). Effect of Ti-OH formation on bioactivity of vacuum plasma sprayed titanium coating after chemical treatment. Surf. Coating Tech..

[40] Takadama H., Kim H.M., Kokubo T., Nakamura T. (2001). XPS study of the process of apatite formation on bioactive Ti-6Al-4V alloy in simulated body fluid. Sci. Tech. Adv. Mater..

[41] Ntais S., Dracopoulos V., Siokou A. (2004). TiCl_4_(THF)_2_ impregnation on a flat SiO_x_/Si(1 0 0) and on polycrystalline Au foil: Determination of surface species using XPS. J. Mol. Catal. A Chem..

[42] Lausmaa J. (1996). Surface spectroscopic characterization of titanium implant materials. J. Electron Spectros. Relat. Phenom..

[43] Feng B., Chen J.Y., QI S.K., He L., Zhao J.Z., Zhang X.D. (2002). Characterization of surface oxide films on titanium and bioactivity. J. Mater. Sci. Mater. Med..

[44] Takeuchi M., Abe Y., Yoshida Y., Nakayama Y., Okazaki M., Akagawa Y. (2003). Acid pretreatment of titanium implants. Biomaterials.

[45] Pouilleau J., Devilliers D., Groult H. (1997). Surface study of a titanium-based ceramic electrode material by X-ray photoelectron spectroscopy. J. Mater. Sci. Eng. A.

[46] Lu G., Bernasek S.L., Schwartz J. (2000). Oxidation of a polycrystalline titanium surface by oxygen and water. Surf. Sci..

[47] Shirkhanzadeh M. (1995). XRD and XPS characterization of superplastic TiO_2_ coatings prepared on Ti6Al4V surgical alloy by an electrochemical method. J. Mater. Sci. Mater. Med..

[48] Sundgren J.E., Bodo P., Lundstrom I. (1986). Auger electron spectroscopic studies of the interface between human tissue and implants of titanium and stainless steel. J. Colloid Interface Sci..

[49] Sham T.K., Lazarus M.S. (1979). X-ray photoelectron spectroscopy (XPS) studies of clean and hydrated TiO_2_ (Rutile) surfaces. Chem. Phys. Lett..

[50] Boehm H.P. (1971). Acidic and basic properties of hydroxylated metal oxide surfaces. Discuss. Faraday Soc..

[51] Kokubo T. (2005). Design of bioactive bone substitutes based on mineralization process. Mater. Sci. Eng. C.

[52] Resende C.X., Dille J., Platt G.M., Bastos N.I., Soares G.A. (2008). Characterization of coating produced on titanium surface by a designed solution containing calcium and phosphate ions. Mater. Chem. Phys..

[53] Kokubo T., Kim H.M., Kawashita M., Nakamura T. (2004). Bioactive metals: Preparation and properties. J. Mater. Sci. Mater. Med..

[54] Wang X.X., Hayakawa S., Tsuru K., Osaka A. (2000). Improvement of bioactivity of H_2_O_2_/TaCl_2_-treated titanium after subsequent heat treatments. J. Biomed. Mater. Res..

[55] Cui X., Kim H.M., Kawashita M., Wang L., Xiong T., Kokubo T., Nakamura T. (2008). Effect of hot water and heat treatment on the apatite-forming ability of titania films formed on titanium metal via anodic oxidation in acetic acid solutions. J. Mater. Sci. Mater. Med..

[56] Takemoto M., Fujibayashi S., Neo M., Suzuki J., Matsushita T., Kokubo T., Nakamura T. (2006). Osteoinductive porous titanium implants: Effect of sodium removal by dilute HCl treatment. Biomaterials.

[57] Barrere F., Snell M.E., van Blitterswijk C.A., De Groot K., Layrolle P. (2004). Nano-scale study of the nucleation and growth of calcium phosphate coating on titanium implants. Biomaterials.

[58] Kokubo T., Kim H.M., Kawashita M. (2003). Novel bioactive materials with different mechanical properties. Biomaterials.

[59] Uchida M., Kim H.M., Fujibayashi S., Nakamura T. (2003). Structural dependence of apatite formation on titania gels in a simulated body fluid. J. Biomed. Mater. Res..

[60] Bigi A., Boanini E.B., Bracci A., Facchini S., Panzavolta F., Segatti L. (2005). Sturba nanocrystalline hydroxyapatite coatings on titanium:a new fast biomimetic method. Biomaterials.

[61] Uchida M., Kim H.M., Kokubo T., Fujibayashi S., Nakamura T. (2002). Effect of water treatment on the apatite-forming ability of NaOH-treated titanium metal. J. Biomed. Mater. Res..

[62] Li F., Feng Q.L., Cui F.Z., Li H.D., Schubert H. (2002). A simple biomimetic method for calcium phosphate coating. Surf. Coating Tech..

[63] Davies J.E. (2007). Bone bonding at natural and biomaterial surfaces. Biomaterials.

[64] Davies J.E. (2003). Understanding peri-implant endosseous healing. J. Dent. Educ..

[65] You C., OH S., Kim S. (2001). Influences of heating condition and substrate-surface roughness on the characteristics of sol-gel-derived hydroxyapatite coatings. J. Sol-Gel Sci. Tech..

[66] Blackwood D.J., Seah K.H. (2009). Seah Influence of anodization on the adhesion of calcium and phosphate coatings on titanium substrates. J. Biomed. Mater. Res..

[67] Liu D.P., Majewski P.J., O'Neill B.K., Ngothai Y., Colby C.B. (2006). The optimal SAM surface functional group for producing a biomimetic HA coating on Ti. J. Biomed. Mater. Res..

[68] Lin C.M., Yen S.K. (2005). Characterization and bond strength of electrolytic HA/TiO_2_ double layers for orthopaedic applications. J. Mater. Sci. Mater. Med..

[69] Koutsopoulos S. (2002). Synthesis and characterization of hydroxyapatite crystals: A review study on the analytical methods. J. Biomed. Mater. Res..

[70] Li H., Huang W., Zhang Y., Zhong M. (2007). Biomimetic synthesis of enamel-like hydroxyapatite on self-assembled monolayers. Mater. Sci. Eng. C.

[71] Yousefpour M., Afashar A., Yang X., Li X., Yang B., Wu Y., Chen J., Zhang X. (2006). Nano-crystalline growth of electrochemically deposited apatite coating on pure titanium. J. Electroanal. Chem..

[72] Leeuwenburgh S., Layrolle P., Barrere F., de Bruijn J., Schoonman J., van Blitterswijk C.A., de Groot K. (2001). Osteoclastic resorption of biomimetic calcium phosphate coatings *in vitro*. J. Biomed. Mater. Res..

[73] He L., Feng Z. (2007). Preparation and characterization of dicalcium phosphate dihydrate coating on enamel. Mater. Lett..

[74] Ermrich M., Peters F. (2006). X-ray powder diffraction data of synthetic b-tricalcium phosphate. Z. Kristallogr. Suppl..

[75] Furuzono T., Walsh D., Yasuda S., Sato K., Tanaka J., Kishida A. (2005). Preparation of plated β-tricalcium phosphate containing hydroxyapatite for use in bonded inorganic-organic composites. J. Mater. Sci..

[76] Rajabi-Zamani A.H., Behnamghader A., Kazemzadeh A. (2008). Synthesis of nanocrystalline carbonated hydroxyapatite powder via nonalkoxide sol-gel method. Mater. Sci. Eng. C.

[77] Kim H.E., Himeno T., Kawashita M., Kokubo T., Nakamura T. (2004). The mechanism of biomineralization of bone-like apatite on synthetic hydroxyapatite: An *in vitro* assessment. J. R. Soc. Interface.

[78] Kim H.M., Miyaji F., Kokubo T. (1997). Effect of heat treatment on apatite-forming ability of Ti metal induced by alkali treatment. J. Mater. Sci. Mater. Med..

[79] Schmidt M. (2001). X-ray photoelectron spectroscopy studies on adsorption of amino acids from aqueous solutions onto oxidised titanium surfaces. Arch. Orthop. Trauma Surg..

[80] Tomazic B., Tomson M., Nancollas G.H. (1975). Growth of calcium phosphates on hydroxyapatite crystals: The effect of magnesium. Arch. Oral. Biol..

[81] Barrere F., van Blitterswijk C.A., de Groot K., Layrolle P. (2002). Nucleation of biomimetic Ca-P coatings on Ti6Al4V from a SBF× 5 solution: influence of magnesium. Biomaterials.

[82] Salimi M.H., Heughebaert J.C., Nancollas G.H. (1985). Crystal growth of calcium phosphates in the presence of magnesium ions. Langmuir.

[83] Rey C., Bracci B., Goehl T., Dickson I.R., Glimcher M.J. (1989). The carbonate environment in bone mineral: A resolution-enhanced fourier transform infrared spectroscopy study. Calcif. Tissue Int..

[84] Vignoles M., Bonel G., Holcomb D.W., Young R.A. (1988). Influence of preparation conditions on the composition of type B carbonated hydroxyapatite and on the localization of the carbonate ions. Calcif. Tissue Int..

[85] Cheng Z.H., Yasukawa A., Kandori K., Ishikawa T. (1998). FTIR study on incorporation of CO_2_ into calcium hydroxyapatite. J. Chem. Soc. Faraday Trans..

[86] Zhang Q., Chen J., Feng J., Cao Y., Deng C., Zhang X. (2003). Dissolution and mineralization behaviours of HA coatings. Biomaterials.

[87] Chang M.C., Douglas W.H., Tanaka J. (2006). Organic-inorganic interaction and the growth mechanism of hydroxyapatite crystals in gelatin matrices between 37 and 80 °C. J. Mater. Sci. Mater. Med..

[88] Stoch A., Jastrzebski W., Brozek A., Trybalska B., Cichocinska M., Szarawara E. (1999). FTIR monitoring of the growth of the carbonate containing apatite layers from simulated and natural body fluids. J. Mol. Struct..

[89] Muller L., Conforto E., Caillard D., Muller F.A. (2007). Biomimetic apatite coatings—Carbonate substitution and preferred growth orientation. Biomol. Eng..

[90] Landi E., Tampieri A., Celotti G., Vichi L., Sandri M. (2004). Influence of synthesis and sintering parameters on the characteristics of carbonate apatite. Biomaterials.

[91] Piotrowski O., Madore C., Landolt D. (1998). The mechanism of electropolishing of titanium in methanol-sulfuric acid electrolytes. J. Electrochem. Soc..

[92] Kim H.M., Miyazaki T., Kokubo T., Nakamura T. (2001). Revised simulated body fluid. Key Eng. Mater..

[93] Kokubo T., Takadama H. (2006). How useful is SBF in predicting *in vivo* bone bioactivity?. Biomaterials.

