# Supplementary material for: Calcium Phosphate Growth at Electropolished Titanium Surfaces
Source: J Funct Biomater. 2012 Apr 25;3(2):327–48. doi: 10.3390/jfb3020327 (PMC4047935; doi:10.3390/jfb3020327)
Supplement: Supplementary File 1 — PDF-Document (PDF, 621 KB) [file jfb-03-00327-s001.pdf]

J. Funct. Biomater. **2012**, *3*

OPEN ACCESS
Journal of
Functional Biomaterials
ISSN 2079-4983
www.mdpi.com/journal/jfb/

Article

Calcium Phosphate Growth at Electropolished Titanium Surfaces

Elnaz Ajami [1,*] and Kondo-Francois Aguey-Zinsou [2]

[1] School of Engineering and Materials Science, University of London, Queen Mary, London E1 4NS, UK
[2] School of Chemical Engineering, The University of New South Wales, Sydney NSW 2052, Australia; E-Mail: f.aguey@unsw.edu.au

* Author to whom correspondence should be addressed; E-Mail: elnaz.ajami@utoronto.ca; Tel.: +61-293-857-970; Fax: +61-293-855-966.

Received: 7 February 2012; in revised form: 21 March 2012 / Accepted: 11 April 2012 / Published: 25 April 2012

Abstract: This work investigated the ability of electropolished Ti surface to induce hydroxyapatite (HA) nucleation and growth *in vitro* via a biomimetic method in Simulated Body Fluid (SBF). The HA induction ability of Ti surface upon electropolishing was compared to that of Ti substrates modified with common chemical methods including alkali, acidic and hydrogen peroxide treatments. Our results revealed the excellent ability of electropolished Ti surfaces in inducing the formation of bone-like HA at the Ti/SBF interface. The chemical composition, crystallinity and thickness of the HA coating obtained on the electropolished Ti surface was found to be comparable to that achieved on the surface of alkali treated Ti substrate, one of the most effective and popular chemical treatments. The surface characteristics of electropolished Ti contributing to HA growth were discussed thoroughly.

Keywords: surface treatment; electropolishing; titanium; hydroxyapatite; biomimetic; biomaterial

Figure S1. XPS narrow-scan spectra of Ti2p for all the substrates.

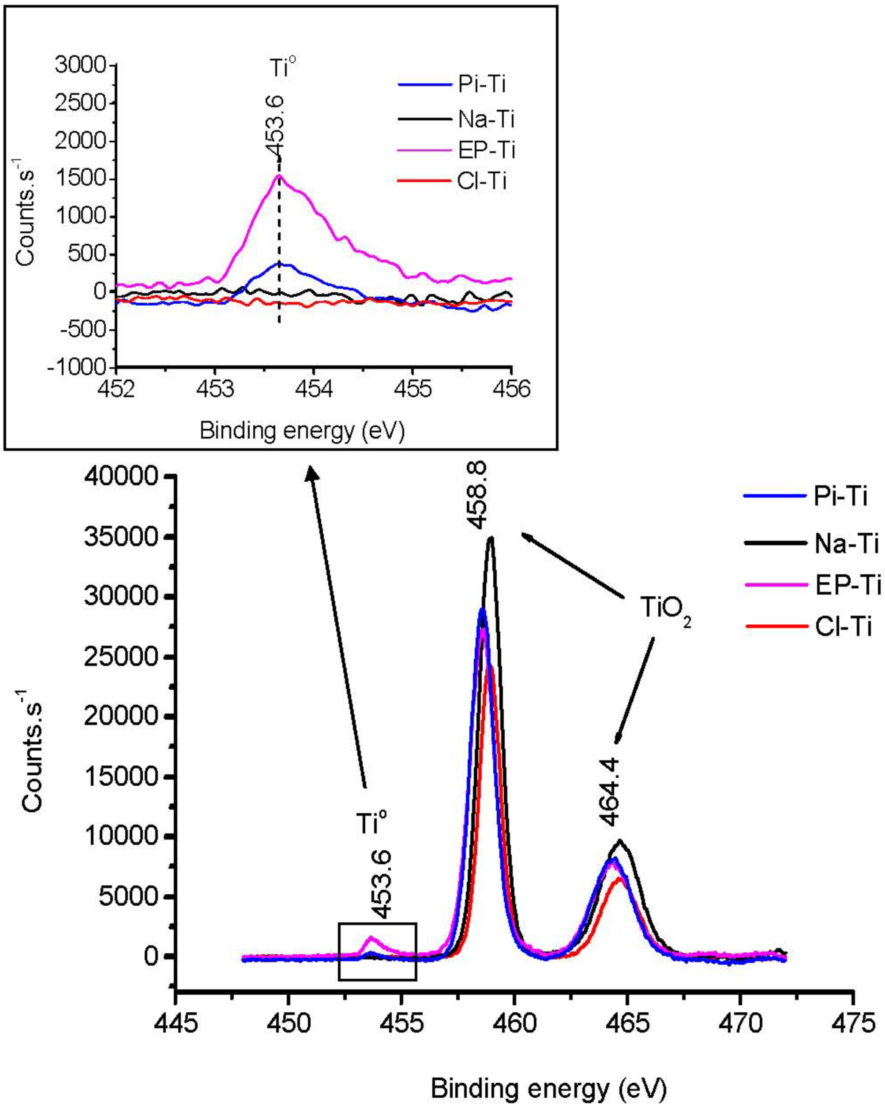

Figure S2. XPS narrow-scan spectra of C1s for all the substrates.

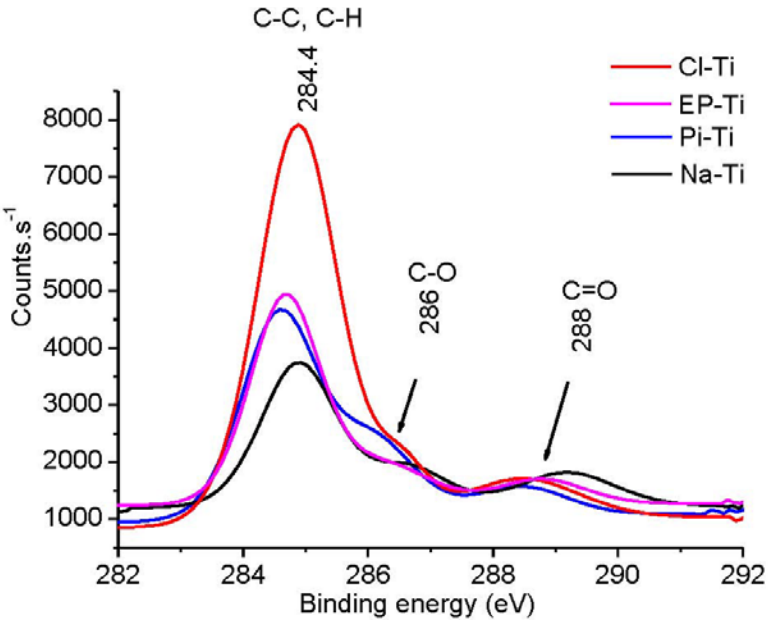

Figure S3. XPS narrow-scan spectra of O1s for the (**a**) EP-Ti; (**b**) Pi-Ti; (**c**) Na-Ti and (**d**) Cl-Ti.

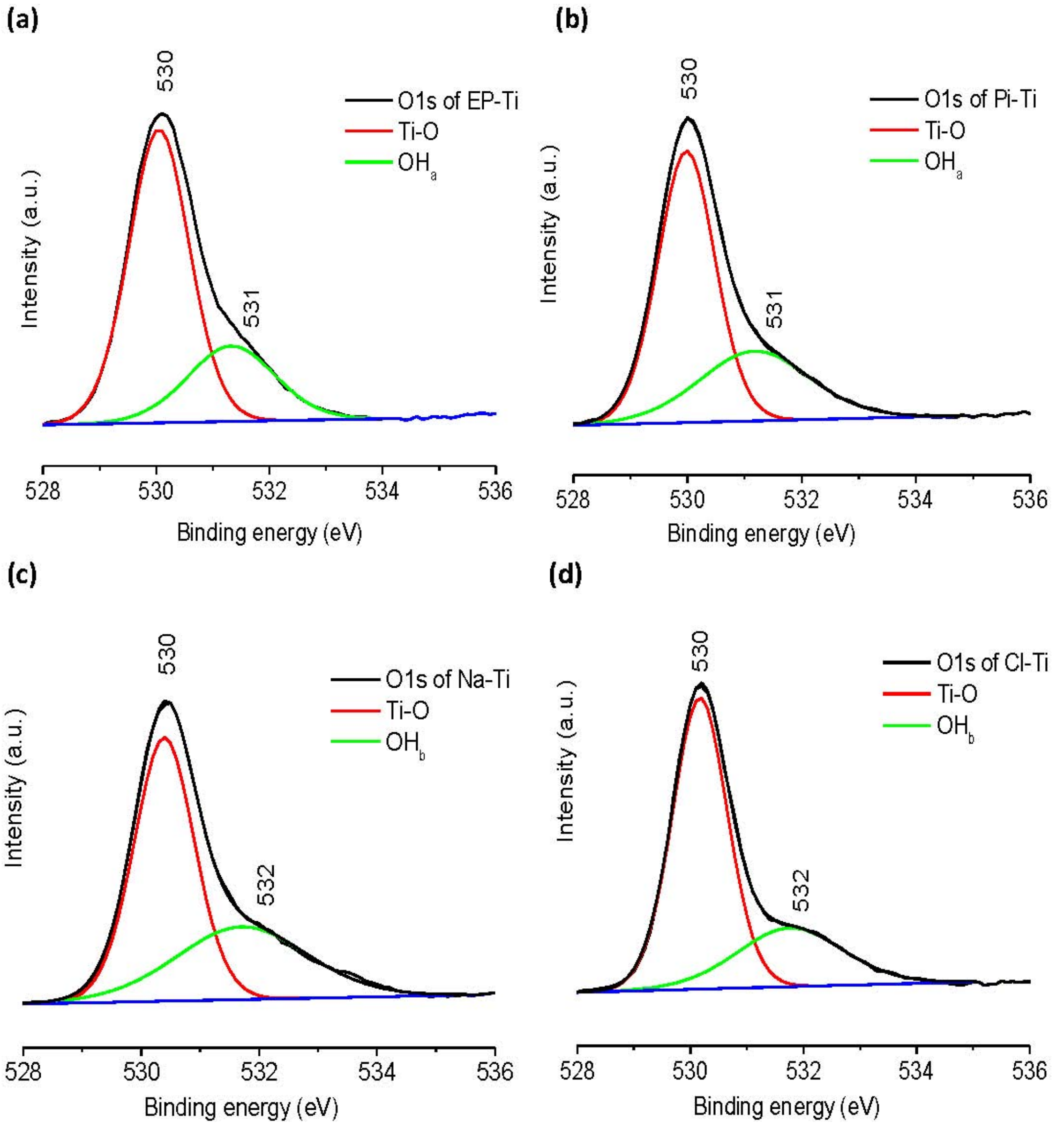

Figure S4. SEM image of plate-like crystals on the CaP globular structures.

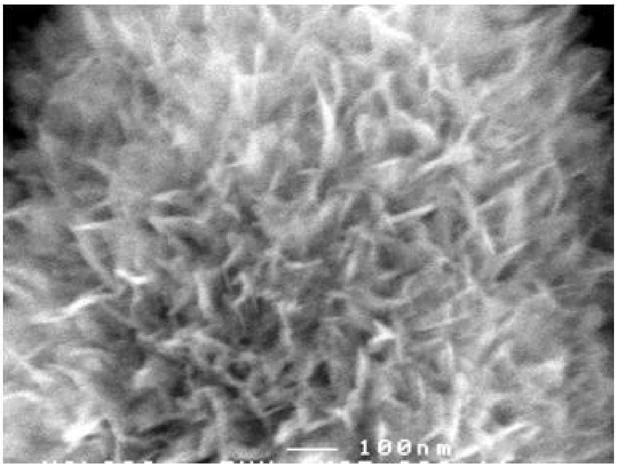

Figure S5. SEM images of Pi-Ti after 1 month immersion in 1.5 SBF.

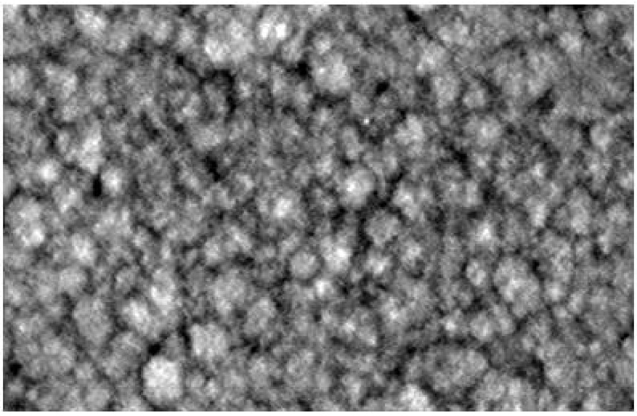

© 2012 by the authors; licensee MDPI, Basel, Switzerland. This article is an open access article distributed under the terms and conditions of the Creative Commons Attribution license (http://creativecommons.org/licenses/by/3.0/).
